# Fabrication and Characterization of Cinnamaldehyde-Loaded Mesoporous Bioactive Glass Nanoparticles/PHBV-Based Microspheres for Preventing Bacterial Infection and Promoting Bone Tissue Regeneration

**DOI:** 10.3390/polym13111794

**Published:** 2021-05-29

**Authors:** Kittipat Chotchindakun, Jeeraporn Pekkoh, Jetsada Ruangsuriya, Kai Zheng, Irem Unalan, Aldo R. Boccaccini

**Affiliations:** 1 Department of Biology, Faculty of Science, Chiang Mai University, Chiang Mai 50200, Thailand; kittipat_ch@cmu.ac.th; 2 Research Center of Microbial Diversity and Sustainable Utilization, Faculty of Science, Chiang Mai University, Chiang Mai 50200, Thailand; 3 Department of Biochemistry, Faculty of Medicine, Chiang Mai University, Chiang Mai 50200, Thailand; jetsada.ruang@cmu.ac.th; 4 Functional Food Research Unit, Science and Technology Research Institute, Chiang Mai University, Chiang Mai 50200, Thailand; 5 Institute of Biomaterials, University of Erlangen-Nuremberg, 91058 Erlangen, Germany; kai.zheng@fau.de (K.Z.); irem.unalan@fau.de (I.U.)

**Keywords:** polyhydroxyalkanoates, bioactive glass nanoparticles, cinnamon oil, emulsion solvent evaporation method, drug delivery systems, antibacterial activity, bone tissue engineering

## Abstract

Polyhydroxybutyrate-co-hydroxyvalerate (PHBV) is considered a suitable polymer for drug delivery systems and bone tissue engineering due to its biocompatibility and biodegradability. However, the lack of bioactivity and antibacterial activity hinders its biomedical applications. In this study, mesoporous bioactive glass nanoparticles (MBGN) were incorporated into PHBV to enhance its bioactivity, while cinnamaldehyde (CIN) was loaded in MBGN to introduce antimicrobial activity. The blank (PHBV/MBGN) and the CIN-loaded microspheres (PHBV/MBGN/CIN5, PHBV/MBGN/CIN10, and PHBV/MBGN/CIN20) were fabricated by emulsion solvent extraction/evaporation method. The average particle size and zeta potential of all samples were investigated, as well as the morphology of all samples evaluated by scanning electron microscopy. PHBV/MBGN/CIN5, PHBV/MBGN/CIN10, and PHBV/MBGN/CIN20 significantly exhibited antibacterial activity against *Staphylococcus aureus* and *Escherichia coli* in the first 3 h, while CIN releasing behavior was observed up to 7 d. Human osteosarcoma cell (MG-63) proliferation and attachment were noticed after 24 h cell culture, demonstrating no adverse effects due to the presence of microspheres. Additionally, the rapid formation of hydroxyapatite on the composite microspheres after immersion in simulated body fluid (SBF) during 7 d revealed the bioactivity of the composite microspheres. Our findings indicate that this system represents an alternative model for an antibacterial biomaterial for potential applications in bone tissue engineering.

## 1. Introduction

The bacterial bone infection, called osteomyelitis, represents a notable healthcare burden [1]. Osteomyelitis is usually treated through systemic drug administration. Conventional antibiotics are a crucial tool against bacterial infection. However, the extensive and redundant antibiotic usage involves bacterial antibiotic resistance consequences from their evolution. It is, therefore, mandatory to examine new antimicrobial approaches to treat infections caused by microorganisms [2].

Since ancient times, several medicinal plants have been employed to treat a variety of diseases. More than 1500 plants have been investigated according to their biological compounds and pharmaceutical properties [3]. Among them, CIN, which is the dominant compound in essential oils obtained from *Cinnamomum* sp., possesses multiple functions in medicinal use [4,5]. CIN’s antimicrobial activity has been demonstrated against a broad range of pathogens, such as *Escherichia coli, Staphylococcus aureus, Klebsiella pneumonia, Pseudomonas aeruginosa*, and *Listeria monocytogenes* [6,7,8]. Moreover, CIN also exhibits a wide range of beneficial biological activities, such as antioxidant, antitumor, and anti-inflammatory activities [9,10]. In the context of osteomyelitis, CIN has also been reported as an alternative to treat the infection from multi-drug resistant (MDR) bacteria [11,12]. However, only a limited amount of the compound can usually be delivered to the infection site, leading thereby to a less effective curing of the disease [13]. Therefore, in situ antibiotic delivery is considered to increase local drug concentration and prolong the release period without adverse effects on the body.

Drug delivery systems (DDS) represent a strategy to carry the required therapeutic agents, including antibiotics, into the target site [14]. The precise selection of the pertinent carrier material is an important consideration when performing DDS. The selected material should be biocompatible and biodegradable, resulting in low or no stimulation of the autoimmune system and without an extended presence in the body. For these reasons, some biodegradable polymers have been recognized as appropriate materials for drug delivery carriers [15]. Among various polymeric biomaterials, polyhydroxybutyrate-co-hydroxyvarelate (PHBV) is an attractive material for use as a drug delivery carrier due to its relevant period biodegradability [16]. The degradable by-product (i.e., 3-hydroxy acids) of PHBV is commonly found in blood, demonstrating its high biocompatibility [17]. PHBV can be fabricated in different forms by various techniques, such as electrospinning technique [18], emulsion freezing/freeze-drying technique [19,20], electrospraying [21,22], and emulsion solvent extraction/evaporation technique [23]. Porous PHBV microspheres can be achieved via emulsion solvent extraction/evaporation technique contributing to unique local site releasing [24]. Over the past decades, PHBV has been widely used in bone tissue engineering [25,26,27]. However, due to its hydrophobic characteristic, the lack of bioactivity, and low cellular interaction, applications of PHBV in bone regeneration are limited [28,29]. The combination of PHBV with other bioactive materials to overcome the limitations mentioned above is expected to promote their potential in bone tissue regeneration [30,31].

Mesoporous bioactive glass nanoparticles (MBGN) are promising biomaterials in various tissue regeneration strategies, particularly for bone tissue regeneration [32]. In regard to microstructure and morphology, MBGN possess a narrow pore size distribution with a significantly improved surface area and pore volume [33]. These properties indicate superior bioactivity and biodegradability. MBGN have also been shown to exhibit osteogenic as well as potential angiogenic effects [34,35,36]. For these reasons, incorporating MBGN into PHBV material can overcome the limitations of PHBV (e.g., lack of bioactivity) and also improve the performance of the polymer as drug delivery system [37].

The present study aimed to develop CIN-loaded MBGN/PHBV-based microspheres as a drug delivery platform for preventing osteomyelitis and enhancing bone tissue regeneration. In this work, we demonstrated the influence of different CIN (5, 10, 20% v/v) concentrations on the properties of composite microspheres, including morphology, releasing behavior, antibacterial activity, and cytotoxicity. The bioactivity of the composite microspheres due to the presence of MBGN was also evidenced. Even if PHBV, MBGN, or CIN alone have exhibited properties useful in bone tissue engineering, their combination for bone regeneration has not been reported previously. We hypothesized that antibacterial activity and enhanced bioactivity of the composite microspheres could be achieved by careful design of their composition and morphology.

## 2. Materials and Methods

### 2.1. Materials

PHBV, with a natural origin and PHV content of 12 mol%, was purchased from Goodfellow (Stadt Bad Nauheim, Germany). CIN and dichloromethane (DCM) were ordered from Sigma-Aldrich (Darmstadt, Germany). Dulbecco’s Phosphate Buffered Saline (DPBS) was purchased from Gibco (Waltham, MA, USA). The microorganism strains of *Staphylococcus aureus* (ATCC25923) and *Escherichia coli* (ATCC25922) were obtained from the Institute of Biomaterials (University of Erlangen-Nuremberg, Erlangen, Germany). Lysogeny broth (LB) medium was supplied by Carl Roth GmbH (Karlsruhe, Germany). Dulbecco’s modified Eagle’s medium (DMEM), fetal bovine serum (FBS), penicillin/streptomycin (PS), and trypsin/EDTA solution were purchased from Thermo Scientific (Schwerte, Germany). MG-63 human osteosarcoma cell line was obtained from Sigma-Aldrich (Cas number: 86051601-1VL, Darmstadt, Germany). All the other chemicals for mesoporous bioactive glass nanoparticle (MBGN) synthesis, fabrication of CIN-loaded PHBV/MBGN microspheres, and simulated body fluid (SBF) preparation were obtained from Merck (Darmstadt, Germany). All chemicals used were of an analytical grade.

### 2.2. Fabrication of CIN-Loaded PHBV/MBGN Microspheres

First of all, MBGN were synthesized by means of a microemulsion-assisted sol-gel process as described elsewhere [38]. The CIN-loaded PHBV microspheres’ particles were fabricated by emulsion solvent extraction/evaporation method, which was modified from a previous study [23]. Briefly, 10% (w/w) of MBGN was added into 3 mL of 3% (w/v) of PHBV in DCM (oil phase). The different concentrations of CIN to PHBV/MBGN-DCM solution were varied in the ratio of 5%, 10%, and 20% (v/v). The components were mixed by a magnetic stirrer (VMS-C4, VWR, Darmstadt, Germany) for 2 h. Then, the homogeneous mixture was added into 75 mL of 2% w/v PVA solution (water phase-1) and emulsified using a homogenizer (T18, IKA, Staufen, Germany) at 7000 rpm for 3 min. Afterwards, the obtained emulsion was added into 225 mL of 1% w/v PVA solution (water phase-2). The final solution was stirred at 600 rpm for 2 h by an overhead stirrer (Eurostar 20, IKA, Staufen, Germany) to evaporate the remaining organic solvent. The microspheres were collected by centrifugation (Centrifuge 5430R, Eppendorf, Hamburg, Germany) at 5000 rpm for 4 min, washed in deionized water, and lyophilized (Alpha 1-2 LDplus, Martin Christ, Osterode am Harz, Germany). The microspheres were coded as PHBV/MBGN/CIN5, PHBV/MBGN/CIN10, and PHBV/MBGN/CIN20 corresponding to the different concentration of CIN, and then kept at −20 °C until further use.

PHBV/MBGN microspheres were prepared as a blank, following the procedure mentioned earlier, with no addition of CIN.

### 2.3. Characterization of CIN-Loaded PHBV/MBGN Microspheres

#### 2.3.1. Scanning Electron Microscopy

The morphology of blank microspheres, i.e., PHBV/MBGN, and CIN-loaded microspheres, i.e., PHBV/MBGN/CIN5, PHBV/MBGN/CIN10, and PHBV/MBGN/CIN20 was characterized using a scanning electron microscopy (SEM) (AURIGA 55, Zeiss, Munich, Germany). The samples were sputter-coated with gold (Q150T plus, Quorum, Laughton, UK) in a vacuum before observation. SEM was also used to observe the microsphere surfaces after SBF immersion, cell proliferation, and cell attachment.

#### 2.3.2. Mean Particle Size and Zeta Potential Analysis

Mean particle size and zeta potential of samples were analyzed by a Zetasizer (Nano ZS, Malvern, Worcestershire, UK). The samples were suspended in deionized water. All measurements were carried out in triplicate.

#### 2.3.3. Fourier Transformed Infrared (FTIR) Spectroscopy

The chemical composition of samples was investigated by a Fourier transformed infrared (FTIR) spectroscopy (Nicolet 6700, Thermo Scientific, Waltham, MA, USA). The spectra were recorded in the absorbance mode in the range of 4000 and 400 cm^−1^ with a resolution of 4 cm^−1^. FTIR was also used to evaluate the chemical structure of the samples after SBF immersion.

#### 2.3.4. X-ray Diffraction (XRD) Analysis

The crystallized structure of microsphere samples after SBF immersion was evaluated by using a X-ray diffractometer (Miniflex 600, Rigaku, Tokyo, Japan). A step size of 0.020° comprised with a dwell time of 1 s per step was used in 2θ ranging from 20° to 60°.

#### 2.3.5. Energy Dispersive X-ray Spectroscopy (EDS) Analysis

The composition of microsphere samples after SBF immersion was analyzed using an energy dispersive spectroscopy (EDS) (X-Max^N^, Oxford Instruments, Abingdon, UK) during SEM imaging.

### 2.4. Encapsulation Efficiency

The CIN encapsulation efficiency of PHBV/MBGN/CIN5, PHBV/MBGN/CIN10, and PHBV/MBGN/CIN20 microspheres was evaluated by the indirect analytical method described in the literature [39]. The amount of unentrapped CIN was used to calculate the total entrapped CIN in PHBV/MBGN microspheres. Briefly, microsphere samples (2 mg) were submerged in 2 mL of methanol (Sigma-Aldrich, Darmstadt, Germany) for obtaining unentrapped CIN. The supernatant was measured through the absorbance UV-vis spectrophotometry at 287 nm (Specord 250, Analytikjena, Jena, Germany). The amount of unentrapped CIN was calculated using the linear regression equation, corresponding to the calibration curve of CIN (Figure S1). Then, the encapsulation efficiency of CIN was calculated as follows:

$$\mathrm{Encapsulation}\;\mathrm{efficiency}\;\left(\%\right)=\frac{\left(CIN_{the}-CIN_{sup}\right)}{CIN_{the}}\times 100$$

where *CIN_sup_* and *CIN_the_* are the supernatant amount and the theoretical amount of CIN (mg) in PHBV/MBGN microspheres, respectively.

### 2.5. In Vitro CIN Releasing Behavior

The in vitro CIN releasing behavior of PHBV/MBGN/CIN5, PHBV/MBGN/CIN10, and PHBV/MBGN/CIN20 microspheres was performed in SBF. Microsphere samples (5 mg) were rinsed with methanol to remove unentrapped CIN. The microsphere samples were placed into sterile 15 mL tubes with caps, each containing 5 mL of SBF (pH = 7.4) as described previously [40]. The tubes were then placed on a shaking incubator (KS 4000i control, IKA, Staufen, Germany) at 37 °C and 75 rpm. To estimate the release of CIN, supernatant volumes were taken at different times for up to 336 h, and replaced with fresh SBF. The CIN releasing was obtained using a UV-vis spectrophotometry absorbance at 293 nm (Specord 250, Analytikjena, Jena, Germany). Then, the actual release of CIN was calculated, corresponding to the calibration curve (Figure S2). All samples were performed in triplicate. The data obtained from in vitro CIN releasing behavior were fitted to various mathematical models, e.g., zero-order, first-order, Higuchi, Hixson-Crowell, Korsmeyer–Peppas, to determine the CIN release kinetics. The fitted model was subsequently selected based on a comparison of the relevant correlation coefficients [41].

### 2.6. Antibacterial Assay

The antibacterial activity of PHBV/MBGN/CIN5, PHBV/MBGN/CIN10, and PHBV/MBGN/CIN20 microspheres was evaluated in *Staphylococcus aureus* (Gram-positive) and *Escherichia coli* (Gram-negative) bacteria. For both bacterial stains, the bacterial suspension was first prepared with LB medium at 37 °C for 24 h. To achieve the initial bacterial suspension, the optical density of 600 nm (Specord 250, Analytikjena, Jena, Germany) was set to 0.015. Microspheres (2 mg) were sterilized with UV light for 30 min before being added to 2 mL of LB medium, and 20 µL of bacterial suspension was applied separately. All samples were incubated at 37 °C. Each time at 3, 6, and 24 h, the absorbance of the supernatant was assessed at 600 nm to determine bacterial viability. The relative viability of bacteria was calculated according to the following equation:

$$\mathrm{Relative}\;\mathrm{bacterial}\;\mathrm{viability}=\frac{absorbance\;of\;sample}{absorbance\;of\;reference}\times 100$$

As a blank and reference, lysogeny broth medium and bacterial cell suspension in the lysogeny broth medium were used, respectively. All experiments were performed in triplicate.

### 2.7. In Vitro Cytotoxicity Test

The cytotoxicity test of PHBV/MBGN/CIN5, PHBV/MBGN/CIN10, and PHBV/MBGN/CIN20 microspheres was carried out using human osteosarcoma MG-63 cells. The cell viability was investigated using a colorimetric (WST-8 based) assay (Steinheim, Germany). MG-63 cells were first cultured in 75 cm^2^ cell culture flasks in DMEM supplemented with 10% FBS, and 1% PS solutions, collected with Trypsin/EDTA solution, and counted with a hemocytometer (Carl Roth GmbH, Karlsruhe, Germany). The microsphere samples were sterilized for 30 min with UV light before being submerged in 48-well plates in different ratios (1000, 100, 10, and 1 µg mL^−1^). The counted cells were seeded at a density of 50,000 cells per well and incubated for 24 h at 37 °C with 5% CO_2_ for 24 h. The cell viability was then determined by incubating them in DMEM with 5% WST-8 reagent at 37 °C for 2 h. Finally, using a spectrophotometric plate reader (PHOmo, Anthos Mikrosysteme GmbH, Friesoythe, Germany), the absorbance of the dye obtained was estimated at 450 nm. The percentage of cell viability was calculated as follows:

$$\mathrm{Cell}\;\mathrm{viability}\;\left(\%\right)=\frac{\left(absorbance\;of\;reference-absorbance\;of\;sample\right)}{\left(absorbance\;of\;reference-absorbance\;of\;blank\right)}\times 100$$

The absorbance of culture medium with cells and WST-8 reagent was used as a control and blank, respectively. Each sample was measured in triplicate.

### 2.8. In Vitro Bioactivity Test

The in vitro bioactivity test (i.e., apatite-forming ability) of CIN-loaded PHBV/MBGN microspheres was carried out by the SBF immersion test as previously reported [40]. Briefly, microspheres were soaked in SBF at a concentration of 1 mg mL^−1^ and kept at 37 °C in a shaking incubator (KS 4000i control, IKA, Staufen, Germany), at 90 rpm for 7 d. After that, the sample was collected and lyophilized (Alpha 1-2 LDplus, Martin Christ, Osterode am Harz, Germany). The in vitro bioactivity, in terms of the formation of hydroxyapatite on the microsphere surfaces, was then characterized by SEM, FTIR, XRD, and EDS.

### 2.9. Statistical Analysis

All experimental data are reported as mean ± standard deviation for at least three independent experiments. The statistical analysis of the data was carried out using one-way analysis of variance (ANOVA) with IBM SPSS Statistics (Version 25; IBM, Armonk, NY, USA). To evaluate the significant difference between groups, Duncan’s new multiple range test was applied [8,42]. The level of the statistical significance was defined as *p* < 0.05.

## 3. Results and Discussion

### 3.1. Characterization of Microspheres

#### 3.1.1. Scanning Electron Microscopy

**Figure 1:**
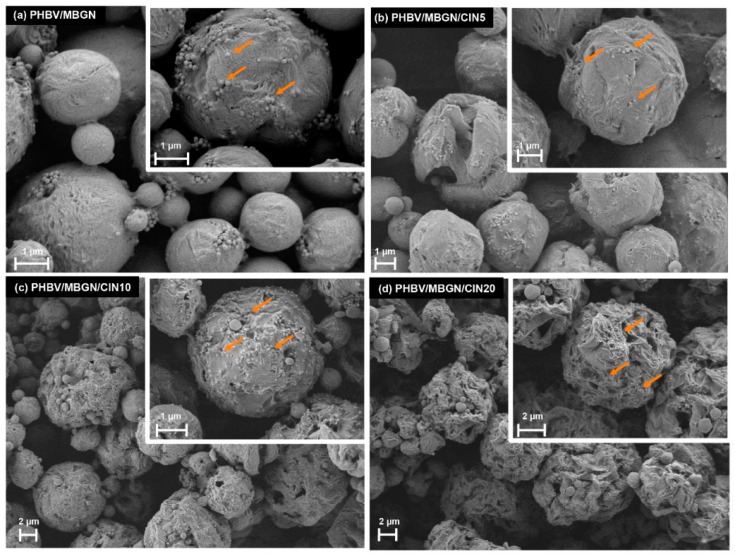
Scanning electron micrographs of (**a**) PHBV/MBGN; (**b**) PHBV/MBGN/CIN5; (**c**) PHBV/MBGN/CIN10, and (**d**) PHBV/MBGN/CIN20 microspheres. Arrows indicate mesoporous bioactive glass nanoparticles.

The morphologies of blank microspheres, i.e., PHBV/MBGN, and CIN-loaded microspheres, i.e., PHBV/MBGN/CIN5, PHBV/MBGN/CIN10, and PHBV/MBGN/CIN20, are shown in Figure 1a–d. A spherical morphology featuring mesoporous bioactive glass nanoparticles on the surface was observed in all samples. A smooth-compact surface with slightly rough structures was observed in the blank microsphere, as previously described [43]. In contrast, apparent rough structures with some visible surface pores were noticed in CIN-loaded microspheres. Furthermore, the higher rough structures correlated with the increase of CIN concentration. The diffusion of CIN during the preparation process could explain this observation. The faster the speed of CIN diffusion, the greater the rough surface of microspheres [44,45]. Moreover, some agglomerates of CIN-loaded microspheres were observed in SEM images, especially in PHBV/MBGN/CIN20. This behavior resulted from the increased surface energy of the system, due to the addition of CIN, thereby reducing the stability of PVA during the preparation process [46].

#### 3.1.2. Particle Size, Polydispersity Index, Zeta Potential, and Encapsulation Efficiency

The average diameter, polydispersity index (PDI), zeta potential, and encapsulation efficiency of PHBV/MBGN, PHBV/MBGN/CIN5, PHBV/MBGN/CIN10, and PHBV/MBGN/CIN20 microspheres are reported in Table 1. The average diameter was measured to be in the range of 6.1 µm to 12.5 µm. A significantly higher average diameter compared to blank PHBV/MBGN microspheres was exhibited by PHBV/MBGN/CIN10, and PHBV/MBGN/CIN20 microspheres (*p* < 0.05). This finding could be related to the increased viscosity of the dispersed phase due to the greater polymer molar mass, leading to harder splitting of the smaller microspheres and thus allowing the formation of large particles [47,48].

PDI values, between 0.4 and 0.9, were shown in all samples. However, a higher PDI value than 0.7, which was only exhibited by blank PHBV/MBGN microspheres, indicates a broad particle size distribution [49] which would hinder the kinetics of drug release due to intra-tissue dispersion and penetration [50,51]. A slight decrease in PDI values compared to blank PHBV/MBGN microspheres was noticed by adding CIN into the microspheres. A significant difference was demonstrated by PHBV/MBGN/CIN5 (*p* < 0.05), indicating that CIN tended to favor the homogeneity of the microsphere size distribution.

Zeta potential values between −21.3 mV and −12.2 mV were measured in all samples. There was no significant difference among the samples except for PHBV/MBGN/CIN20 microspheres (*p* < 0.05). The negative zeta potential of microspheres was obtained due to terminal carboxylic groups (–COOH) in the PHBV matrix. The significant decrease of zeta potential value of PHBV/MBGN/CIN20, compared to blank PHBV/MBGN microspheres, could be explained by the excess adsorption of CIN on their surfaces. These findings agree with previous reports in that several therapeutic agents can mask –COOH groups resulting in a reduction of zeta potential values [46,52,53].

High encapsulation efficiencies of 99.96 ± 0.01%, 99.83 ± 0.02%, and 99.26 ± 0.04% were observed in CIN concentration at 5%, 10%, and 20% (v/v), respectively. CIN and PHBV display the same hydrophobic characteristic, which results from functional groups of aromatic aldehyde [54] and extra methyl group (CH3), respectively. Therefore, both of them generally demonstrate a favorable miscibility [55]. The encapsulation efficiency in the present study is similar to that of sunitinib-loaded PHBV microspheres (~94%) produced by emulsion solvent extraction/evaporation method [56], and is higher than that of therapeutic agents with loaded PHBV microspheres produced by electrospraying method (~30−40%) [57], and nanoprecipitation method (~65−70%) [46]. However, a significant reduction in encapsulation efficiency (*p* < 0.05) of PHBV/MBGN microspheres was detected in a further amount of CIN, i.e., 30%, 40%, and 50% (v/v) (Figure S3). Similar behavior has been noticed in other studies upon loading antimicrobial agents into PHBV suggesting that PHBV’s encapsulation efficiency decreases when the loaded material’s concentration is increased [46,58]. Furthermore, greater agglomeration and morphological changes on further CIN concentration were observed by SEM (Figure S4). Therefore, PHBV/MBGN/CIN5, PHBV/MBGN/CIN10, and PHBV/MBGN/CIN20 microspheres were chosen for further studies due to their high encapsulation efficiency and their spherical shape morphology.

#### 3.1.3. FTIR Analysis

**Figure 2:**
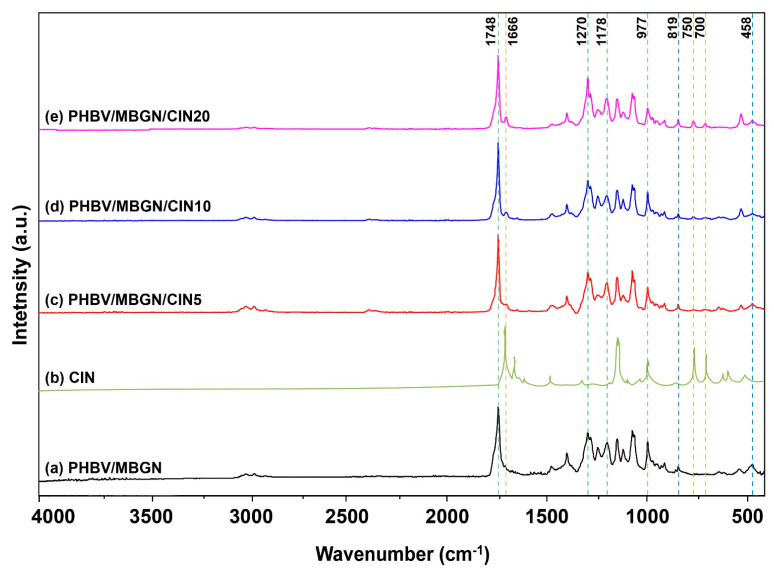
FTIR spectra of (**a**) PHBV/MBGN; (**b**) CIN; (**c**) PHBV/MBGN/CIN5; (**d**) PHBV/MBGN/CIN10, and (**e**) PHBV/MBGN/CIN20 in the range of 4000–400 cm^−1^. The relevant peaks are discussed in the text.

The FTIR spectra of PHBV/MBGN, PHBV/MBGN/CIN5, PHBV/MBGN/CIN10, and PHBV/MBGN/CIN20 microspheres are shown in Figure 2. The bands corresponding to PHBV and MBGN were observed in all samples. The adsorption bands at 977 cm^−1^, 1178 cm^−1^, and 1270 cm^−1^ can be assumed to be the anti-symmetric vibration of C–O–C stretching in the PHBV matrix; also, the band at 1748 cm^−1^ can be referred to the ester carbonyl group (C=O) [59], while the bands at 458 cm^−1^ and 819 cm^–−1^, which are assigned as Si-O-Si bending and symmetric stretching vibration, respectively, indicate the incorporation of MBGN into the PHBV matrix [60]. The additional CIN in the ratios of 5%, 10%, and 20% (v/v) into PHBV/MBGN microspheres was observed with the gradient intensity at the band 1666 cm^−1^ suggested as the carbonyl group (C=O) [61], along with the bands at 700 cm^−1^ and 750 cm^−1^ consisted of CH=CH bending in alkenes and –CH bending in the aromatic ring [54]. These findings confirm the successful incorporation of MBGN and CIN into PHBV-based microspheres.

### 3.2. In Vitro CIN Release Behavior

**Figure 3:**
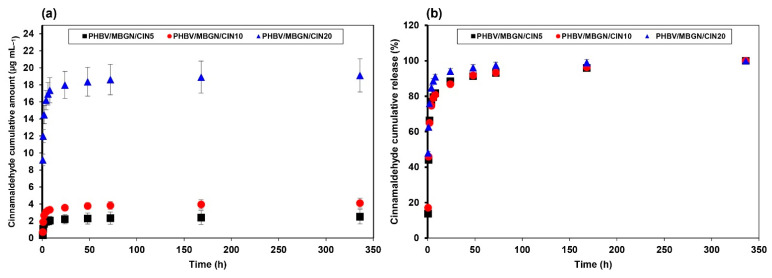
In vitro CIN release behavior of PHBV/MBGN/CIN5, PHBV/MBGN/CIN10, and PHBV/MBGN/CIN20 microspheres in simulated body fluid: (**a**) CIN cumulative amount (µg mL^−1^), and (**b**) CIN cumulative release (%). Experimental data are reported as mean ± standard deviation. *n* = 3. A sample of PHBV/MBGN/CIN20 showed the highest CIN cumulative amount and CIN cumulative release.

CIN cumulative amount (µg mL^−1^) and CIN cumulative release (%) from PHBV/MBGN/CIN5, PHBV/MBGN/CIN10, and PHBV/MBGN/CIN20 microspheres are represented in Figure 3a,b. The level of cumulative CIN (µg mL^−1^) was escalated depending on the initial amount of CIN loaded in microspheres. The highest CIN cumulative amount at 19.11 µg mL^−1^ was noticed in PHBV/MBGN/CIN20 followed by PHBV/MBGN/CIN10 and PHBV/MBGN/CIN5 at 4.11 µg mL^−1^ and 2.52 µg mL^−1^, respectively. The complete release of cumulative CIN (%) of all samples was achieved within 336 h. The release behavior could be separated into two kinetic phases corresponding to their cumulative release. A rapid release was observed within the first 2 h. Approximate 65–75% of total CIN release was achieved in the first-kinetic phase. Further release of CIN in the second-kinetic phase was then slow and gradual. It should be noted that all samples were washed to eliminate the remaining CIN on their surface. Therefore, the initial fast release of CIN (60–75%) was likely due to the CIN entrapped on the semi-porous surface of microspheres. The micro-, nano-porous structure of PHBV and MBGN exposed high-surface areas in contact with the fluid, consequently bursting the CIN release. The gradual release in the later phase resulted from the diffusion through deeper layers of the polymer matrix or possibly due to the depletion of CIN. This behavior was similar not only to PHBV but also to other polyesters, such as polyhydroxybutyrate (PHB)-based microspheres [39,62,63,64] and polylactic-co-glycolic acid (PLGA)-based microspheres [65,66].

In order to understand the pattern of CIN release from PHBV/MBGN microspheres, mathematical kinetic models, including zero order, first order, Higuchi, Hixson-Crowell, and Korsmeyer–Peppas models, were used to predict the kinetic of CIN release (Table 2). Among these kinetic models, the first-order model and the Korsmeyer–Peppas model were suggested as fitting models to explain the release behavior of CIN from PHBV/MBGN microspheres. The n value obtained from the Korsmeyer–Peppas model was less than 0.45, suggesting that a Fickian-diffusion mechanism occurred in the system [67]. Both kinetic models indicated a CIN release mechanism principally followed by a concentration-dependent process [46].

### 3.3. Antibacterial Activity

**Figure 4:**
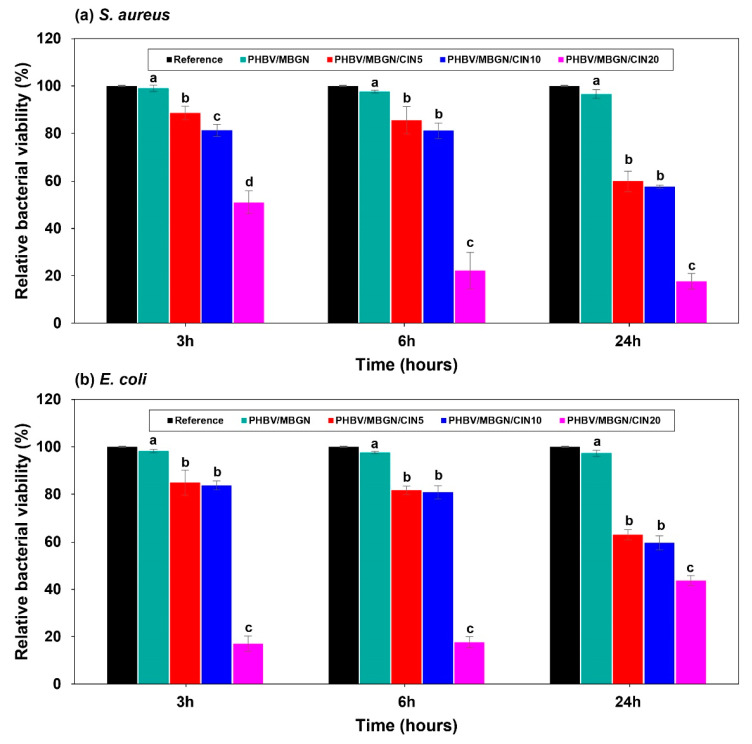
Relative viability of (**a**) *Staphylococcus aureus* and (**b**) *Escherichia coli* on different concentrations of CIN-loaded PHBV/MBGN microspheres (PHBV/MBGN/CIN5, PHBV/MBGN/CIN10, and PHBV/MBGN/CIN20) at 3, 6, and 24 h incubation. Bacterial viability in free microspheres’ medium was used as reference. Experimental data are reported as mean ± standard deviation. *n* = 3. Means followed by the different letters within columns indicate a significant difference at *p* < 0.05 using Duncan’s new multiple range test. Each different time point was analyzed separately.

The relative bacterial viability of *Staphylococcus aureus* and *Escherichia coli* on the different CIN-loaded PHBV/MBGN microspheres is represented in Figure 4a,b. The investigation was carried out at 3, 6, and 24 h during the incubation period. The presence of blank PHBV/MBGN microspheres led to no significant difference (*p* < 0.05) in both bacterial strain’s bacterial viability compared to the reference. This result is in agreement with literature data in that there is no significant antibacterial activity of both PHBV [68,69] and MBGN [70,71]. On the contrary, a significant decrease of the relative bacterial viability on both bacterial strains was shown in PHBV/MBGN/CIN5, PHBV/MBGN/CIN10, and PHBV/MBGN/CIN20, compared to the bacterial viability in free microsphere medium considered as reference (*p* < 0.05). The enhancing antibacterial activity was noticed with increasing CIN concentration. The PHBV/MBGN/CIN20 presented the lowest relative bacterial viability at 17.6% within 24 h for *S. aureus*, and at 17.0% within 3 h for *E. coli*. These findings suggest that the addition of CIN into PHBV/MBGN microspheres led to antibacterial activity against both bacterial strains. However, although CIN-loaded PHBV/MBGN microspheres, especially PHBV/MBGN/CIN20, affected *S. aureus* bacteria even after 24 h, its effect on *E. coli* bacterium started to decrease. These results indicate that CIN has a bactericidal effect on *E. coli.* The bactericidal effect of CIN toward *E. coli* (Gram-negative bacterium) at 24 h could be explained by the presence of the complex double membrane comprised of an extra outer lipopolysaccharide layer in Gram-negative bacteria. These structures can prevent those bacteria from the invasion of antibacterial compounds [72,73,74]. Hence, the reduced efficiency in CIN-loaded PHBV/MBGN microsphere against *E. coli* in comparison to *S. aureus* (Gram-positive bacterium) should be noted. A similar result was reported in the case of cinnamon oil-loaded PLA (polylactic acid) electrospun nanofibrous film [75], and also when considering other essential oils and antibiotic drugs [46,76].

### 3.4. In Vitro Cytotoxicity and Cell Adhesion Assay

**Figure 5:**
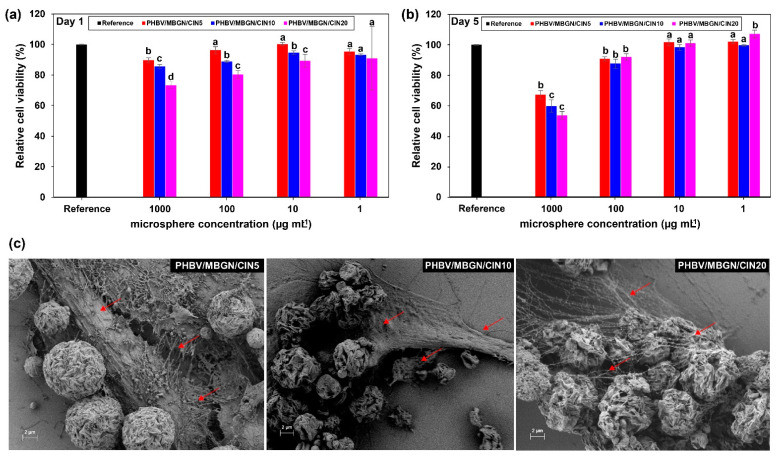
Cytotoxicity test of osteosarcoma MG-63 cells on the different microsphere concentrations (1000, 100, 10, and 1 µg mL^−1^) for (**a**) day 1 and (**b**) day 5. The relative cell viability in free microsphere medium was used as reference. Experimental data are reported as mean ± standard deviation (*n* = 3). Means followed by the different letters within columns indicate a significant difference at *p* < 0.05 using Duncan’s new multiple range test. Each different microsphere concentration was analyzed separately; (**c**) Scanning electron micrographs showing cell adhesion and attachment on the surface of PHBV/MBGN/CIN5, PHBV/MBGN/CIN10, and PHBV/MBGN/CIN20 microspheres at the highest concentration of microspheres, i.e., 1000 µg mL^−1^, after 5 d of culture, the arrows indicate the presence of cells or filopodia.

The viability of human osteosarcoma MG-63 cells was used to evaluate the biocompatibility of PHBV/MBGN/CIN5, PHBV/MBGN/CIN10, and PHBV/MBGN/CIN20. The different doses of microspheres (1000, 100, 10, and 1 µg mL^−1^) and different culture times (1 d and 5 d) were employed to determine possible cytotoxic effects. The relative cell viability is represented in Figure 5. After 1 d of culture, the decline of relative cell viability was revealed in all microsphere concentrations except for microsphere concentration at 1 µg mL^−1^, compared to the cell-free microsphere medium used as reference (Figure 5a). A further significant decrease of relative cell viability was induced with increasing CIN. This finding indicates that those microspheres hindered cell proliferation during the first day of incubation. However, after 5 d of culture, the relative cell viability became higher. The results thus showed no significant differences (*p* < 0.05), especially in the 10 µg mL^−1^ and 1 µg mL^−1^ microsphere concentrations, compared to the cell-free microsphere medium used as reference (Figure 5b). In contrast, significant hindrances were observed for 1000 µg mL^−1^ and 100 µg mL^−1^ microspheres’ concentrations (*p* < 0.05). This behavior could be explained by the “burst effect” of CIN release from microspheres. The high amount of CIN in the first period of culture could interrupt cell proliferation. This evidence is in agreement with previous studies reporting that high concentrations of CIN exhibited cytotoxicity to several cell lines, including osteosarcoma MG-63 cells [77,78]. However, cell proliferation in the initial cell culture was hindered by the presence of PHBV/MBGN/CIN5, PHBV/MBGN/CIN10, and PHBV/MBGN/CIN20 microspheres. It should be noted that all microspheres at all doses exhibited no adverse effects on the proliferation of MG-63 cells during the investigated culture time.

The cell adhesion assay was performed to assess cell adhesion and attachment on the surface of PHBV/MBGN/CIN5, PHBV/MBGN/CIN10, and PHBV/MBGN/CIN20 microspheres at the highest concentration of microspheres (i.e., 1000 µg mL^−1^). After 5 d of culture, all samples were covered by the forming network of cells (Figure 5C). Elongated morphology of cells was also observed, indicating cell growth. This evidence suggested that the surface of PHBV/MBGN/CIN5, PHBV/MBGN/CIN10, and PHBV/MBGN/CIN20 microspheres can facilitate the adhesion and attachment of human osteosarcoma MG-63 cells.

### 3.5. In Vitro Bioactivity

**Figure 6:**
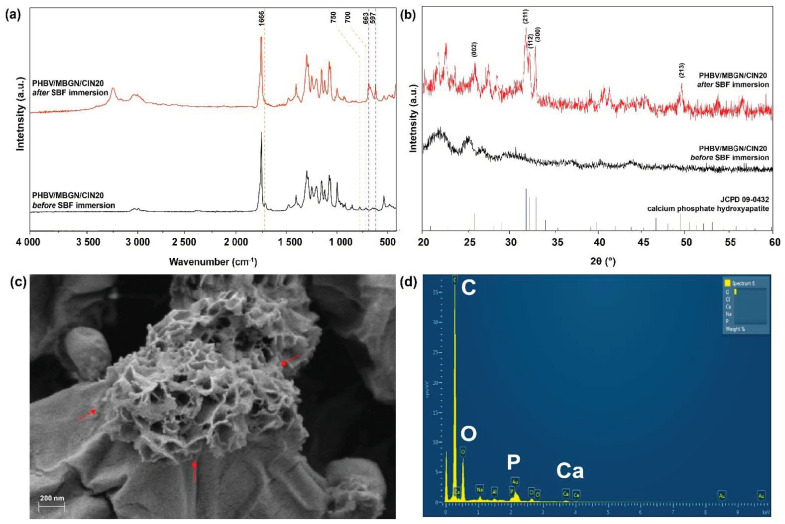
The bioactivity analysis of PHBV/MBGN/CIN20 microspheres by simulated body fluid (SBF) immersion test: (**a**) FTIR spectra; (**b**) XRD patterns; (**c**) Scanning electron micrograph indicating hydroxyapatite formation on the surface of microspheres, as shown by arrows; (**d**) A representative EDS spectrum showing the presence of C, O, P, and Ca. The peak of Au is detected from the substrate as a result of the gold sputtering process.

PHBV/MBGN/CIN20 microspheres were chosen as a representative sample due to the measured highest antibacterial activity coupled with no adverse effects on the attachment of MG-63 cells, corresponding to the antibacterial and cell adhesion assays. The results after immersion in SBF for 7 d are shown in Figure 6. The two new bands, located at 597 cm^−1^ and 663 cm^−1^, were observed in the FTIR spectrum of PHBV/MBGN/CIN20 (Figure 6a), which could correspond to the P-O bending vibrations in crystalline hydroxyapatite (HA) [23,79]. Further Si-O-Si bonds, observed at 458 cm^−1^ and 819 cm^−1^, indicate the presence of MBGN in the microspheres. Additionally, the typical CIN bands at 700 cm^−1^, 750 cm^−1^, and 1666 cm^−1^ exhibit a weak signal. This behavior is associated with the release of CIN from PHBV/MBGN microspheres. The new diffraction signals of the XRD pattern (Figure 6b) were detected at approximate by 2θ = 26.0°, 31.7°, 32.1°, 32.9°, and 49.4°, which correspond to hydroxyapatite (JCPD 09-0432) [80]. Besides, with the observation of surface morphology (Figure 6c), needle-like structures were seen to be present on the surface of PHBV/MBGN/CIN20 microspheres, which exhibit the characteristic morphology of HA formed on MBGN after immersing in SBF [70,81]. Further verification of the presence of HA was performed by EDS analysis (Figure 6d). The EDS spectrum of SBF immersed PHBV/MBGN/CIN20 microspheres confirmed the presence of Ca and P [82]. The peak of C corresponds to the polymeric matrix (PHBV) [69,83]. These results confirm the bioactivity of MBGN incorporated in PHBV/MBGN/CIN20 microspheres. This finding thus shows the potential use of CIN-loaded PHBV/MBGN microspheres in bone tissue regeneration.

## 4. Conclusions

In this study, CIN-loaded PHBV/MBGN microspheres at different CIN concentrations, i.e., 5%, 10%, and 20% (v/v), were successfully fabricated by the emulsion solvent extraction/evaporation method. The spherical morphology of PHBV/MBGN microspheres was preserved after the incorporation of CIN. A high CIN encapsulation efficiency (≥99%) of PHBV/MBGN microspheres was observed, up to 20% (v/v) of CIN concentration. All samples exhibited antimicrobial activity against *S. aureus* and *E. coli*, especially PHBV/MBGN/CIN20 microspheres were highly antibacterial. Additionally, the incorporation of CIN did not imprint MG-63 cell proliferation and attachment. The CIN-loaded PHBV/MBGN microspheres also exhibited high bioactivity showing rapid hydroxyapatite formation in SBF. Our results thus suggest that CIN-loaded PHBV/MBGN microspheres have potential as antibiotic-free material in drug delivery applications and bone tissue engineering.

## Figures and Tables

**Table 1:** Diameter, polydispersity index (PDI), zeta potential, and encapsulation efficiency of blank microspheres (PHBV/MBGN) and CIN-loaded microspheres ranging from 5 to 20% (v/v) (PHBV/MBGN/CIN5, PHBV/MBGN/CIN10, and PHBV/MBGN/CIN20).

Samples	Diameter (µm)	PDI	Zeta Potential (mV)	Encapsulation Efficiency (%)
PHBV/MBGN	6.1 ± 0.7 ^a^	0.9 ± 0.1 ^a^	−20.7 ± 0.4 ^a^	–
PHBV/MBGN/CIN5	7.2 ± 1.5 ^a^	0.4 ± 0.1 ^b^	−21.3 ± 0.5 ^a^	99.96 ± 0.01 ^a^
PHBV/MBGN/CIN10	11.4 ± 1.6 ^b^	0.6 ± 0.2 ^ab^	−20.4 ± 0.5 ^a^	99.83 ± 0.02 ^a^
PHBV/MBGN/CIN20	12.5 ± 2.3 ^b^	0.5 ± 0.2 ^ab^	−12.2 ± 2.7 ^b^	99.26 ± 0.04 ^a^

Experimental data are reported as mean ± standard deviation. *n* = 3. Means followed by the different letters within columns indicate a significant difference at *p* < 0.05 using Duncan’s new multiple range test. Each column was analyzed separately.

**Table 2:** Mathematical kinetic models applied to in vitro CIN cumulative release (%) of PHBV/MBGN/CIN5, PHBV/MBGN/CIN10, and PHBV/MBGN/CIN20 microspheres in simulated body fluid (*n* = 3).

Systems		Kinetic Models					
	Zero-Order	First Order		Higuchi	Hixson-Crowell	Korsmeyer–Peppas	
	R^2^	R^2^	K1 h^−^^1^	R^2^	R^2^	R^2^	n
PHBV/MBGN/CIN5	−5.22	0.87	0.42	−2.24	−3.01	0.68	0.12
PHBV/MBGN/CIN10	−5.63	0.84	0.41	−2.42	−3.24	0.72	0.12
PHBV/MBGN/CIN20	−18.29	0.76	0.93	−9.95	−12.41	0.74	0.08

## Data Availability

The data presented in this study are available on request from the corresponding author.

## Supplementary Materials

The following are available online at https://www.mdpi.com/article/10.3390/polym13111794/s1, Figure S1: Linear regression equation, corresponding to the calibration curve of cinnamaldehyde in methanol by means of the absorbance UV-vis spectrophotometry at 287 nm, ranging from 0.25 µg mL^−^^1^ to 3.5 µg mL^−^^1^, Figure S2: Linear regression equation, corresponding to the calibration curve of cinnamaldehyde in simulated body fluid (SBF) by means of the absorbance UV-vis spectrophotometry at 293 nm, ranging from 0.25 µg mL^−^^1^ to 3.5 µg mL^−^^1^, Figure S3: Encapsulation efficiency of PHBV/MBGN microspheres as function of CIN concentration ranging from 5 to 50% (v/v), Figure S4: Scanning electron micrographs of (**a**) PHBV/MBGN/CIN30, (**b**) PHBV/MBGN/CIN40, and (**c**) PHBV/MBGN/CIN50 microspheres, Table S1: The raw data of diameter, polydispersity index and zeta potential of blank and cinnamaldehyde-loaded microspheres, Table S2: The raw data of encapsulation efficiency of PHBV/MBGN microspheres on cinnamaldehyde concentration ranging from 5% (v/v) to 50% (v/v), Table S3: The raw data of in vitro cinnamaldehyde cumulative release (%) of PHBV/MBGN/CIN5, PHBV/MBGN/CIN10, and PHBV/MBGN/CIN20 microspheres in phosphate buffer solution within 336 h, Table S4: The raw data of Dissolution Data Modeling and goodness-of-fit of zero order model, Table S5: The raw data of Dissolution Data Modeling and goodness-of-fit of first order model, Table S6: The raw data of Dissolution Data Modeling and goodness-of-fit of Higuchi model, Table S7: The raw data of Dissolution Data Modeling and goodness-of-fit of Hixson model, Table S8: The raw data of Dissolution Data Modeling and goodness-of-fit of Korsmeyer–Peppas model, Table S9: The raw data of antibacterial activity of PHBV/MBGN/CIN5, PHBV/MBGN/CIN10, and PHBV/MBGN/CIN20 microspheres on *S. aureus* and *E. coli*, Table S10: The raw data of cytotoxicity test of PHBV/MBGN/CIN5, PHBV/MBGN/CIN10, and PHBV/MBGN/CIN20 microspheres on osteosarcoma MG-63 cells.
