# Supplementary material for: Fabrication and Characterization of Cinnamaldehyde-Loaded Mesoporous Bioactive Glass Nanoparticles/PHBV-Based Microspheres for Preventing Bacterial Infection and Promoting Bone Tissue Regeneration
Source: Polymers (Basel). 2021 May 29;13(11):1794. doi: 10.3390/polym13111794 (PMC8198921; doi:10.3390/polym13111794)
Supplement: Supplementary file 1 [file polymers-13-01794-s001.zip › polymers-1209013-supplementary.pdf]

Supplementary material S1

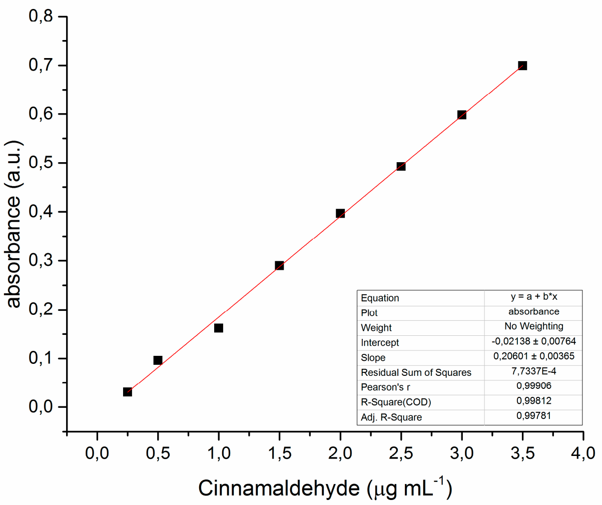

Figure S1. Linear regression equation, corresponding to the calibration curve of cinnamaldehyde in methanol by means of the absorbance UV-vis spectrophotometry at 287 nm, ranging from 0.25 µg mL^{-1} to 3.5 µg mL^{-1}

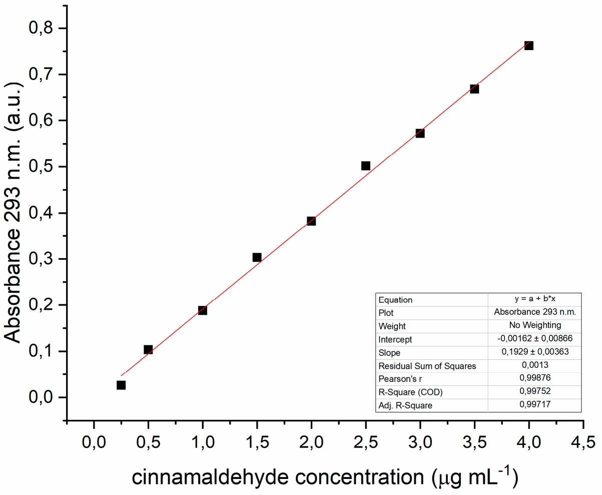

Figure S2. Linear regression equation, corresponding to the calibration curve of cinnamaldehyde in simulated body fluid (SBF) by means of the absorbance UV-vis spectrophotometry at 293 nm, ranging from 0.25 µg mL^{-1} to 3.5 µg mL^{-1}

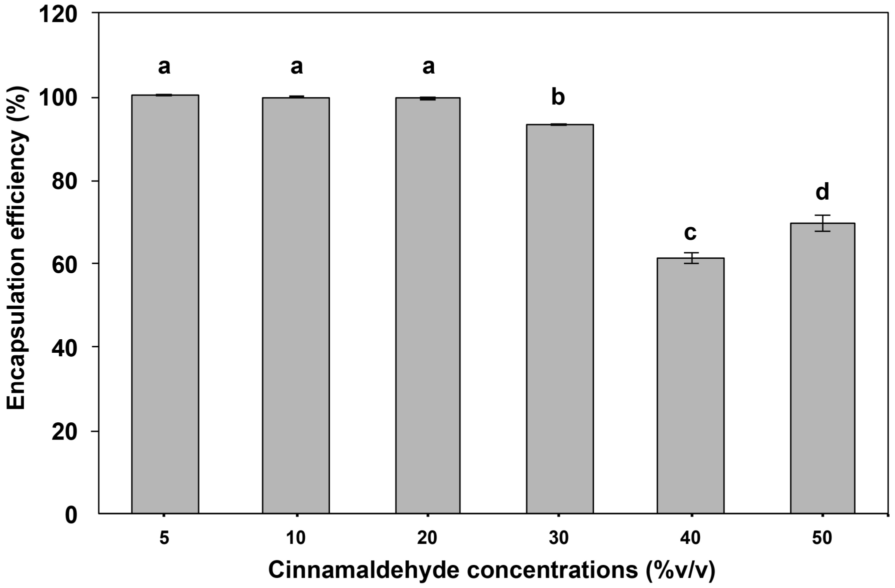

Figure S3. Encapsulation efficiency of PHBV/MBGN microspheres as function of CIN concentration ranging from 5 to 50% (v/v). Experimental data are reported as mean ± standard deviation. $n = 3$. Means followed by the different letters within columns indicate a significant difference at $p < 0.05$ using Duncan's new multiple range test.

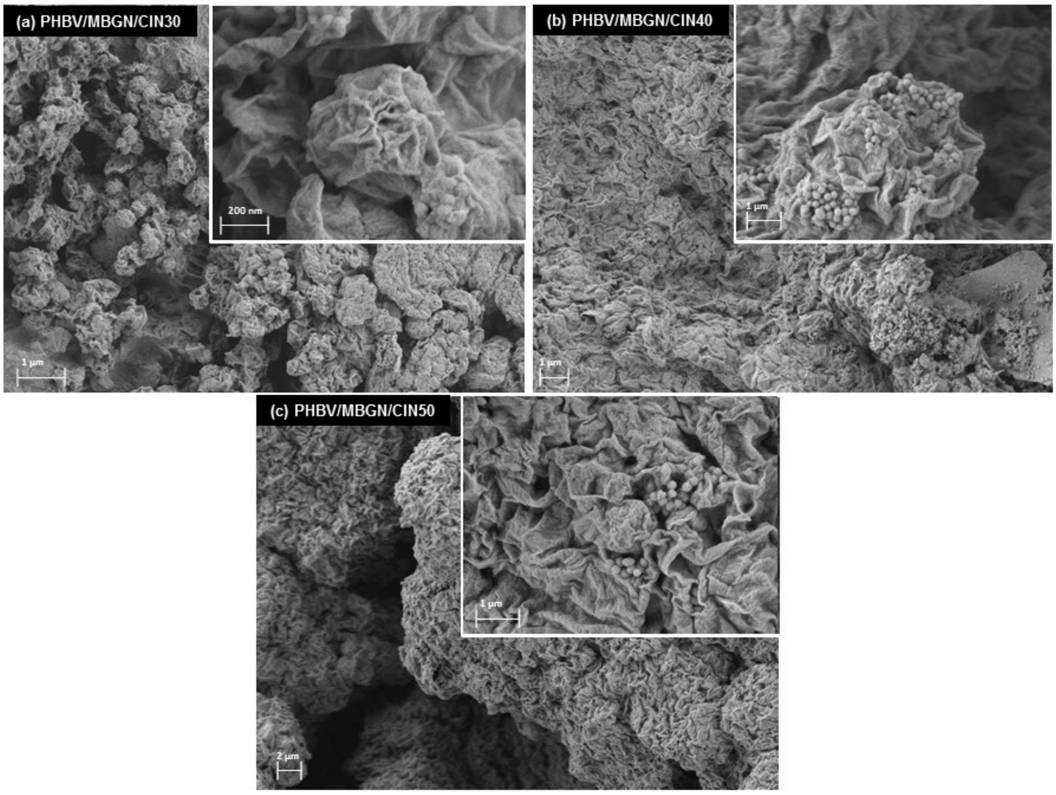

Figure S4. Scanning electron micrographs of (a) PHBV/MBGN/CIN30, (b) PHBV/MBGN/CIN40, and (c) PHBV/MBGN/CIN50 microspheres.

Table S1. the raw data of diameter, polydispersity index and zeta potential of blank and cinnamaldehyde-loaded microspheres.

sample	Size (μm)					PDI					Zeta potential (mV)				
	#1	#2	#3	AVG	STV	#1	#2	#3	AVG	STV	#1	#2	#3	AVG	STV
PHBV/MBGN	6.8	5.1	6.5	6.1	0.7	1.0	0.7	0.9	0.9	0.1	-20.2	-21.2	-20.8	-20.7	0.4
PHBV/MBGN/CIN5	5.9	6.2	9.3	7.2	1.5	0.5	0.4	0.4	0.4	0.1	-20.7	-21.4	-21.8	-21.3	0.5
PHBV/MBGN/CIN10	10.4	10.0	13.6	11.4	1.6	0.6	0.3	0.8	0.6	0.2	-20.1	-21.1	-20.0	-20.4	0.5
PHBV/MBGN/CIN20	13.7	14.5	9.2	12.5	2.3	0.6	0.8	0.2	0.5	0.2	-15.9	-10.0	-10.6	-12.2	2.7

Table S2. the raw data of encapsulation efficiency of PHBV/MBGN microspheres on cinnamaldehyde concentration ranging from 5 % (v/v) to 50% (v/v)

Cinnamaldehyde concentration (% v/v)	Encapsulation efficiency (%)				
	#1	#2	#3	AVG	STV
5%	99.9635	99.9563	99.9579	99.9592[a]	0.0031
10%	99.8503	99.8481	99.7997	99.8327[a]	0.0233
20%	99.2044	99.3072	99.2678	99.2598[a]	0.0423
30%	55.8538	48.3190	49.0014	51.0581[b]	3.4025
40%	60.5063	62.8804	60.1217	61.1695[c]	1.2199
50%	66.4520	70.4902	71.6485	69.5303[d]	2.2274

Table S3. the raw data of *in vitro* cinnamaldehyde cumulative release (%) of PHBV/MBGN/CIN5, PHBV/MBGN/CIN10, and PHBV/MBGN/CIN20 microspheres in phosphate buffer solution within 336 h

The different time point (h)	sample	Cinnamaldehyde cumulative release (μg mL^{-1})				
		#1	#2	#3	AVG	STV
0.5	PHBV/MBGN/CIN5	0.20	0.43	0.40	0.35	0.10
0.5	PHBV/MBGN/CIN10	0.79	0.77	0.56	0.71	0.11
0.5	PHBV/MBGN/CIN20	9.12	10.04	8.39	9.19	0.68
1	PHBV/MBGN/CIN5	0.87	1.29	1.18	1.11	0.18
1	PHBV/MBGN/CIN10	1.90	1.99	1.78	1.89	0.09
1	PHBV/MBGN/CIN20	11.97	12.91	11.04	11.97	0.76
2	PHBV/MBGN/CIN5	1.32	1.99	1.70	1.67	0.27
2	PHBV/MBGN/CIN10	2.65	2.78	2.58	2.67	0.08
2	PHBV/MBGN/CIN20	14.41	15.86	13.23	14.50	1.08
4	PHBV/MBGN/CIN5	1.54	2.36	1.90	1.94	0.33
4	PHBV/MBGN/CIN10	3.06	3.23	2.93	3.07	0.12
4	PHBV/MBGN/CIN20	15.97	17.70	14.95	16.21	1.14
6	PHBV/MBGN/CIN5	1.54	2.56	1.90	2.00	0.42
6	PHBV/MBGN/CIN10	3.21	3.48	3.05	3.25	0.18
6	PHBV/MBGN/CIN20	16.61	18.71	15.53	16.95	1.32
8	PHBV/MBGN/CIN5	1.54	2.73	1.90	2.06	0.50
8	PHBV/MBGN/CIN10	3.21	3.71	3.05	3.32	0.28
8	PHBV/MBGN/CIN20	16.94	19.37	15.83	17.38	1.48
24	PHBV/MBGN/CIN5	1.66	2.98	2.03	2.23	0.55
24	PHBV/MBGN/CIN10	3.43	4.02	3.24	3.57	0.33
24	PHBV/MBGN/CIN20	17.45	20.16	16.36	17.99	1.60
48	PHBV/MBGN/CIN5	1.66	3.19	2.03	2.30	0.65
48	PHBV/MBGN/CIN10	3.60	4.28	3.43	3.77	0.37
48	PHBV/MBGN/CIN20	17.76	20.69	16.66	18.37	1.70

72	PHBV/MBGN/CIN5	1.66	3.34	2.03	2.34	0.72
72	PHBV/MBGN/CIN10	3.60	4.47	3.43	3.83	0.46
72	PHBV/MBGN/CIN20	17.98	21.07	16.83	18.62	1.79
168	PHBV/MBGN/CIN5	1.66	3.54	2.03	2.41	0.81
168	PHBV/MBGN/CIN10	3.60	4.72	3.56	3.96	0.54
168	PHBV/MBGN/CIN20	18.18	21.49	17.05	18.91	1.88
336	PHBV/MBGN/CIN5	1.79	3.72	2.03	2.52	0.86
336	PHBV/MBGN/CIN10	3.72	4.94	3.68	4.11	0.59
336	PHBV/MBGN/CIN20	18.33	21.79	17.22	19.11	1.95

Table S4. the raw data of Dissolution Data Modeling and goodness of fit of zero order model

**Parameters**	**#1**	**#2**	**#3**
N_observed	11	11	11
DF	10	10	10
R_obs-pre	0.5184	0.5426	0.5116
Rsqr	-5.2249	-5.6337	-18.2879
Rsqr_adj	-5.2249	-5.6337	-18.2879
MSE	4157.6866	4093.0410	5355.4208
MSE_root	64.4801	63.9769	73.1807
Weighting	1	1	1
SS	41576.8665	40930.4099	53554.2084
WSS	41576.8665	40930.4099	53554.2084
AIC	118.9883	118.8159	121.7729
MSC	-2.0104	-2.0740	-3.1413

Table S5. the raw data of Dissolution Data Modeling and goodness of fit of first order model

**Parameters**	**#1**	**#2**	**#3**
N_observed	11	11	11
DF	10	10	10
R_obs-pre	0.9655	0.9645	0.9617
Rsqr	0.8680	0.8432	0.7773
Rsqr_adj	0.8680	0.8432	0.7773
MSE	88.1826	96.7320	61.8218
MSE_root	9.3906	9.8352	7.8627
Weighting	1	1	1
SS	881.8260	967.3205	618.2179
WSS	881.8260	967.3205	618.2179
AIC	76.6019	77.6198	72.6953
MSC	1.8429	1.6711	1.3203

Table S6. the raw data of Dissolution Data Modeling and goodness of fit of Higuchi model

**Parameters**	**#1**	**#2**	**#3**
N_observed	11	11	11
DF	10	10	10
R_obs-pre	0.6667	0.6929	0.6707
Rsqr	-2.2395	-2.4213	-9.9452
Rsqr_adj	-2.2395	-2.4213	-9.9452
MSE	2163.7177	2110.9723	3039.0105

MSE_root	46.5158	45.9453	55.1272
Weighting	1	1	1
SS	21637.1769	21109.7227	30390.1052
WSS	21637.1769	21109.7227	30390.1052
AIC	111.8039	111.5324	115.5406
MSC	-1.3572	-1.4118	-2.5747

Table S7. the raw data of Dissolution Data Modeling and goodness of fit of Hixson model

**Parameters**	**#1**	**#2**	**#3**
N_observed	11	11	11
DF	10	10	10
R_obs-pre	0.6665	0.6955	0.6737
Rsqr	-3.0093	-3.2426	-12.4093
Rsqr_adj	-3.0093	-3.2426	-12.4093
MSE	2677.8719	2617.7142	3723.1951
MSE_root	51.7482	51.1636	61.0180
Weighting	1	1	1
SS	26778.7195	26177.1422	37231.9512
WSS	26778.7195	26177.1422	37231.9512
AIC	114.1490	113.8991	117.7741
MSC	-1.5704	-1.6270	-2.7778

Table S8. the raw data of Dissolution Data Modeling and goodness of fit of Korsmeyer-Peppas model

**Parameters**	**#1**	**#2**	**#3**
N_observed	11	11	11
DF	9	9	9
R_obs-pre	0.8271	0.8497	0.8605
Rsqr	0.6812	0.7192	0.7394
Rsqr_adj	0.6457	0.6880	0.7105
MSE	236.6112	192.5241	80.3846
MSE_root	15.3822	13.8753	8.9657
Weighting	1	1	1
SS	2129.5004	1732.7166	723.4613
WSS	2129.5004	1732.7166	723.4613
AIC	88.3001	86.0319	76.4245
MSC	0.7795	0.9064	0.9813

Table S9. the raw data of antibacterial activity of PHBV/MBGN/CIN5, PHBV/MBGN/CIN10, and PHBV/MBGN/CIN20 microspheres on *S. aureus* and *E. coli*

The different time point (h)	sample	Relative bacterial viability (%)				
		#1	#2	#3	AVG	STV
S. aureus						
3	PHBV/MBGN	99.10	99.40	98.60	99.03	0.40
3	PHBV/MBGN/CIN5	83.20	89.60	92.90	88.57	4.93
3	PHBV/MBGN/CIN10	79.00	80.90	83.90	81.27	2.47
3	PHBV/MBGN/CIN20	50.90	55.80	46.20	50.97	4.80
6	PHBV/MBGN	98.30	97.20	97.40	97.63	0.59
6	PHBV/MBGN/CIN5	84.60	91.70	80.10	85.47	5.85
6	PHBV/MBGN/CIN10	77.40	82.30	83.70	81.13	3.31
6	PHBV/MBGN/CIN20	13.40	24.90	28.00	22.10	7.69
24	PHBV/MBGN	98.60	94.90	96.10	96.53	1.89
24	PHBV/MBGN/CIN5	56.00	58.90	64.50	59.80	4.32
24	PHBV/MBGN/CIN10	57.40	58.10	56.90	57.47	0.60
24	PHBV/MBGN/CIN20	20.80	17.80	14.20	17.60	3.30
E. coil						
3	PHBV/MBGN	99.20	97.10	98.30	98.20	1.05
3	PHBV/MBGN/CIN5	88.70	87.10	78.90	84.90	5.26
3	PHBV/MBGN/CIN10	85.20	81.60	84.40	83.73	1.89
3	PHBV/MBGN/CIN20	19.80	17.70	13.60	17.03	3.15
6	PHBV/MBGN	98.30	98.10	97.50	97.97	0.42
6	PHBV/MBGN/CIN5	81.90	79.90	83.30	81.70	1.71
6	PHBV/MBGN/CIN10	81.20	77.80	83.30	80.77	2.78
6	PHBV/MBGN/CIN20	19.90	17.70	15.30	17.63	2.30
24	PHBV/MBGN	98.30	97.70	95.90	97.30	1.25
24	PHBV/MBGN/CIN5	62.60	65.20	61.10	62.97	2.07
24	PHBV/MBGN/CIN10	56.20	62.10	60.20	59.50	3.01
24	PHBV/MBGN/CIN20	45.80	43.50	41.70	43.67	2.06

Table S10. the raw data of cytotoxicity test of PHBV/MBGN/CIN5, PHBV/MBGN/CIN10, and PHBV/MBGN/CIN20 microspheres on osteosarcoma MG-63 cells

The different dose (μg mL^{-1})	sample	Relative cell viability (%)				
		#1	#2	#3	AVG	STV
Day 1						
1000	PHBV/MBGN/CIN5	87.41	90.23	91.35	89.66	1.66
1000	PHBV/MBGN/CIN10	85.15	87.41	84.59	85.71	1.22
1000	PHBV/MBGN/CIN20	72.91	76.35	70.94	73.40	2.24
100	PHBV/MBGN/CIN5	98.68	93.05	96.99	96.24	2.36
100	PHBV/MBGN/CIN10	87.97	89.10	89.66	88.91	0.70
100	PHBV/MBGN/CIN20	83.74	79.31	77.83	80.30	2.51
10	PHBV/MBGN/CIN5	100.38	99.25	101.50	100.38	0.92
10	PHBV/MBGN/CIN10	93.61	96.43	93.61	94.55	1.33
10	PHBV/MBGN/CIN20	85.22	87.68	95.07	89.33	4.19
1	PHBV/MBGN/CIN5	98.12	94.74	93.05	95.30	2.11
1	PHBV/MBGN/CIN10	92.48	93.05	94.17	93.23	0.70

**1**	PHBV/MBGN/CIN20	87.19	67.49	118.23	90.97	20.89
**Day 5**						
**1000**	PHBV/MBGN/CIN5	70.77	64.20	66.70	67.22	2.71
**1000**	PHBV/MBGN/CIN10	56.37	65.45	57.62	59.81	4.02
**1000**	PHBV/MBGN/CIN20	50.51	56.63	53.95	53.70	2.51
**100**	PHBV/MBGN/CIN5	88.94	91.44	92.07	90.81	1.35
**100**	PHBV/MBGN/CIN10	87.37	91.41	84.24	87.68	2.95
**100**	PHBV/MBGN/CIN20	94.13	92.98	89.16	92.09	2.13
**10**	PHBV/MBGN/CIN5	104.28	101.77	98.96	101.67	2.17
**10**	PHBV/MBGN/CIN10	100.84	98.02	96.14	98.33	1.93
**10**	PHBV/MBGN/CIN20	99.49	99.87	104.08	101.15	2.08
**1**	PHBV/MBGN/CIN5	103.03	100.21	103.34	102.19	1.41
**1**	PHBV/MBGN/CIN10	99.90	98.96	100.21	99.69	0.53
**1**	PHBV/MBGN/CIN20	104.85	105.99	110.59	107.14	2.48
